# Ferroptosis in Haematological Malignancies: From Regulatory Networks to Novel Therapeutic Opportunities

**DOI:** 10.1111/jcmm.70790

**Published:** 2025-09-11

**Authors:** Maryam Shayanmanesh, Seyed Esmaeil Ahmadi, Ali Amini

**Affiliations:** ^1^ Department of Hematology and Blood Banking, Faculty of Allied Medicine Iran University of Medical Sciences Tehran Iran; ^2^ Department of Endocrinology, Diabetology, and Clinical Nutrition University Hospital Zurich (USZ) and University of Zurich (UZH) Zurich Switzerland

**Keywords:** ferroptosis, ferroptosis inducers, haematological malignancies, leukaemia, lymphoma

## Abstract

Haematological malignancies encompass a wide spectrum of blood cell disorders with diverse prognoses. Despite recent advances in therapy, many of these disorders remain incurable or exhibit relapse and drug resistance. Ferroptosis, an iron‐dependent form of cell death caused by lipid peroxidation, holds promise as a strategy to overcome the resistance seen with conventional therapies. This review aims to succinctly outline current research concerning the regulatory function of ferroptosis‐related genes (FRGs) and various types of non‐coding RNAs (ncRNAs), as well as various types of ferroptosis inducers (FINs), encompassing small molecule compounds, natural derivatives, synthetic agents and nanoparticles. The exploration delves into the mechanisms by which FINs operate, including inhibiting the system Xc^−^, deactivating the enzyme glutathione peroxidase 4 (GPX4), disrupting glutathione (GSH) production and interfering with iron or lipid metabolism. The investigation emphasises the functioning of these agents and the underlying molecular processes driving the initiation of ferroptosis. A comprehensive assessment reveals the potential utility of FINs as innovative treatments for haematological neoplasms, offering insights into a novel therapeutic approach.

## 1 Introduction

Haematological neoplasms arise from malignant transformations of myeloid and lymphoid cells originating from bone marrow (BM) and primary/secondary lymphoid tissues involved in blood production. Common subtypes of BM‐derived malignancies include acute myeloid leukaemia (AML), chronic myeloid leukaemia (CML) and acute lymphoblastic leukaemia (ALL), while chronic lymphocytic leukaemia (CLL), Hodgkin and non‐Hodgkin lymphoma (HL and NHL) and multiple myeloma (MM) emerge from blood or lymphatic organs [1]. A major challenge in treating these malignancies is the frequent evasion of apoptotic cell death, leading to chemotherapeutic resistance and disease recurrence [2].

Cell death incidence is an inevitable endpoint to maintaining physiological homeostasis by eliminating damaged, specifically cancerous cells [3]. Ferroptosis, a subtype of regulated cell death (RCD), also termed programmed cell death (PCD) acts in this regard and now is being used to improve the suboptimal outcomes of conventional antitumor therapies [4, 5, 6]. Therefore, the application of ferroptosis inducers (FINs) represents a promising approach to limiting the survival of malignant cells [7].

The term ‘ferroptosis’ was first minted by Dixon et al. [8] to describe a form of cell death distinct from apoptosis, characterised by its reliance on elevated intracellular iron levels. Ferroptosis is driven by an iron‐catalysed lipid peroxidation process. Unlike established modes of cell death, ferroptosis is triggered by the reactive oxygen species (ROS) reaction with cell membrane polyunsaturated fatty acids (PUFAs); therefore, it leads to membrane breakdown, displaying distinct morphological variations [8, 9]. The main characteristics of ferroptosis encompass a reduction in the size of mitochondria, an increase in membrane density, as well as a reduction/disappearance of mitochondrial cristae [10].

The purpose of this review is to provide an overview of the research on FINs and their underlying mechanisms. Specifically, we will investigate the potential of FINs (small molecules, natural derivative compounds, synthetic agents and nanoparticles) to inhibit the progression of haematological malignancies, as well as look into how ferroptosis‐related genes (FRGs) and non‐coding RNAs (ncRNAs) such as circular RNAs (circRNAs) and long ncRNAs (lncRNAs) are involved. We hope to gain a better understanding of the contribution of ferroptosis to preventing the progression of these particular types of cancer.

## 2 Overviews of Ferroptosis Prerequisites

The occurrence of ferroptosis is linked to various biological processes such as iron metabolism, amino acid metabolism, lipid metabolism, the mevalonate (MVA) pathway, the p53 axis and several other pathways [11, 12, 13]. Ferroptosis‐stimulating agents can impact cellular pathways either internally or externally. Several of them have the capability to impede the exchange of cystine and glutamate amino acids via the Xc^−^ antiporter system or modulate cellular iron levels, ultimately affecting the extrinsic pathway. In contrast, the intrinsic ferroptosis pathway can be triggered through the inhibition of glutathione peroxidase 4 (GPX4), a pivotal player in antioxidant protection [14].

Despite the essential role of iron in signalling, cell proliferation and oxidative phosphorylation, as well as its function as a cofactor in DNA synthesis and repair, both iron deficiency and excess can disrupt normal physiological activities [15, 16]. The transferrin (Tf) molecule brings in Fe^3+^, which is then internalised in a Tf‐Fe^3+^/Tf Receptor 1 (TfR1) complex [17]. Following this, ferric is reduced to ferrous via a six‐transmembrane epithelial antigen of the prostate 3 (STEAP3) ferrireductase enzyme [18]. Furthermore, the reduced form of non‐transferrin‐bound iron (NBTI) can access the cell via transporters such as solute carrier family (SLC) 11 member 2 (SLC11A2), SLC39A8, also known as ZIP8, or SLC39A14, also referred to as ZIP14. On the other side, heme iron has entered the cell via specific heme transporter proteins, including heme carrier protein 1 (HCP1), also known as SLC46A1, heme responsive gene 1 (HRG1), alternatively labelled as SLC48A1, and feline leukaemia virus subgroup C receptor 2 (FLVCR2) [19]. Ultimately, the iron is liberated into the labile iron pool (LIP), which remains metabolically active [20]. LIP is composed of a limited quantity of free or loosely bound intracellular Fe^2+^. The iron shows redox activity, enabling it to engage in Fenton‐type reactions [21, 22]. Furthermore, the autophagy process promotes iron accumulation in LIP through the lysosomal degradation of ferritin (ferritinophagy), which is facilitated by nuclear receptor coactivator 4 (NCOA4) [23, 24]. Then, the non‐enzymatic Fenton reaction between Fe^2+^ and H_2_O_2_ yields the formation of hydroxyl radical (HO^•^), a highly reactive oxidant that particularly targets PUFA and triggers the onset of lipid peroxidation [25]. The hallmarks of ferroptosis are considered excessive ROS production and lipid peroxidation, as they lead to cell membrane permeability and destruction [17].

Besides the non‐enzymatic initiation of lipid peroxidation, enzymatic pathways further amplify this process. In this signalling pathway, the regulation of lipid metabolism is a decisive factor in the occurrence of ferroptosis. The activities of acyl‐CoA synthetase long‐chain family member 4 (ACSL4), phospholipid (PL) remodelling enzyme lysophosphatidylcholine acyltransferase 3 (LPCAT3) and lipoxygenases (LOXs) are involved in generating lipid peroxidation from the primary reservoir of PLs, predominantly consisting of PUFAs [26, 27, 28]. The pro‐ferroptotic ACSL4 enzyme catalyses the formation of long‐chain fatty acid‐CoA species, such as arachidonic acid and adrenic acid (AA‐CoA/AdA‐CoA), for the subsequent formation of phospholipid hydroperoxide (PL‐OOH) [27]. Furthermore, it has been recently discovered that propionate, a short‐chain fatty acid, also participates in ACSL4‐mediated ferroptosis [29]. In the next step, the LPCAT3 enzyme incorporates the acetylated groups into the phosphatidylcholine (PC) or phosphatidylethanolamine (PE) of the membrane to assemble PUFA‐PLs [27, 30]. Ultimately, LOXs that contain non‐heme iron can also accelerate lipid peroxidation through the enzymatic pathway [31].

**FIGURE 1:**
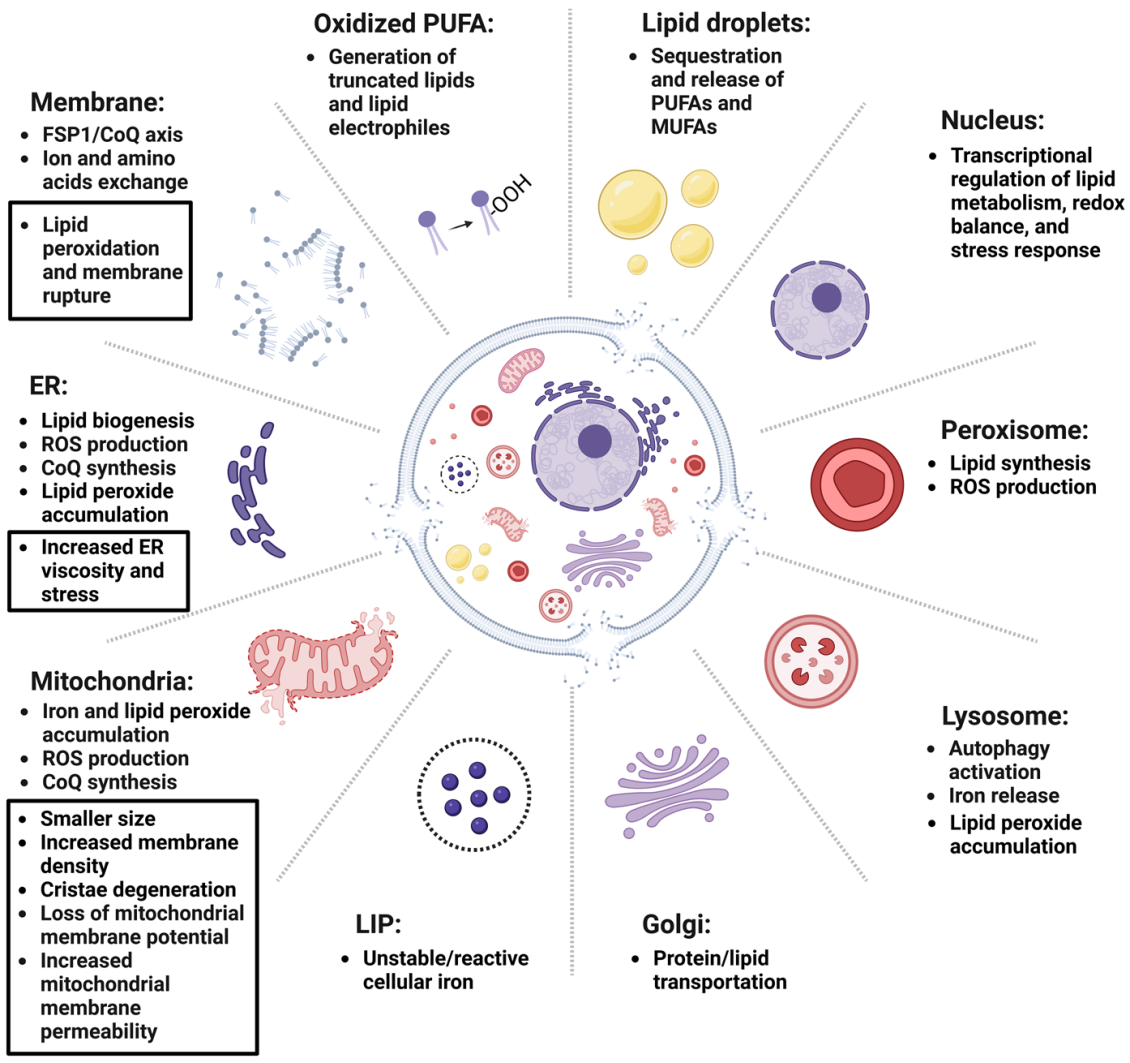
The role of subcellular structures in different pathways influencing ferroptosis and its impact on these organelles.

**FIGURE 2:**
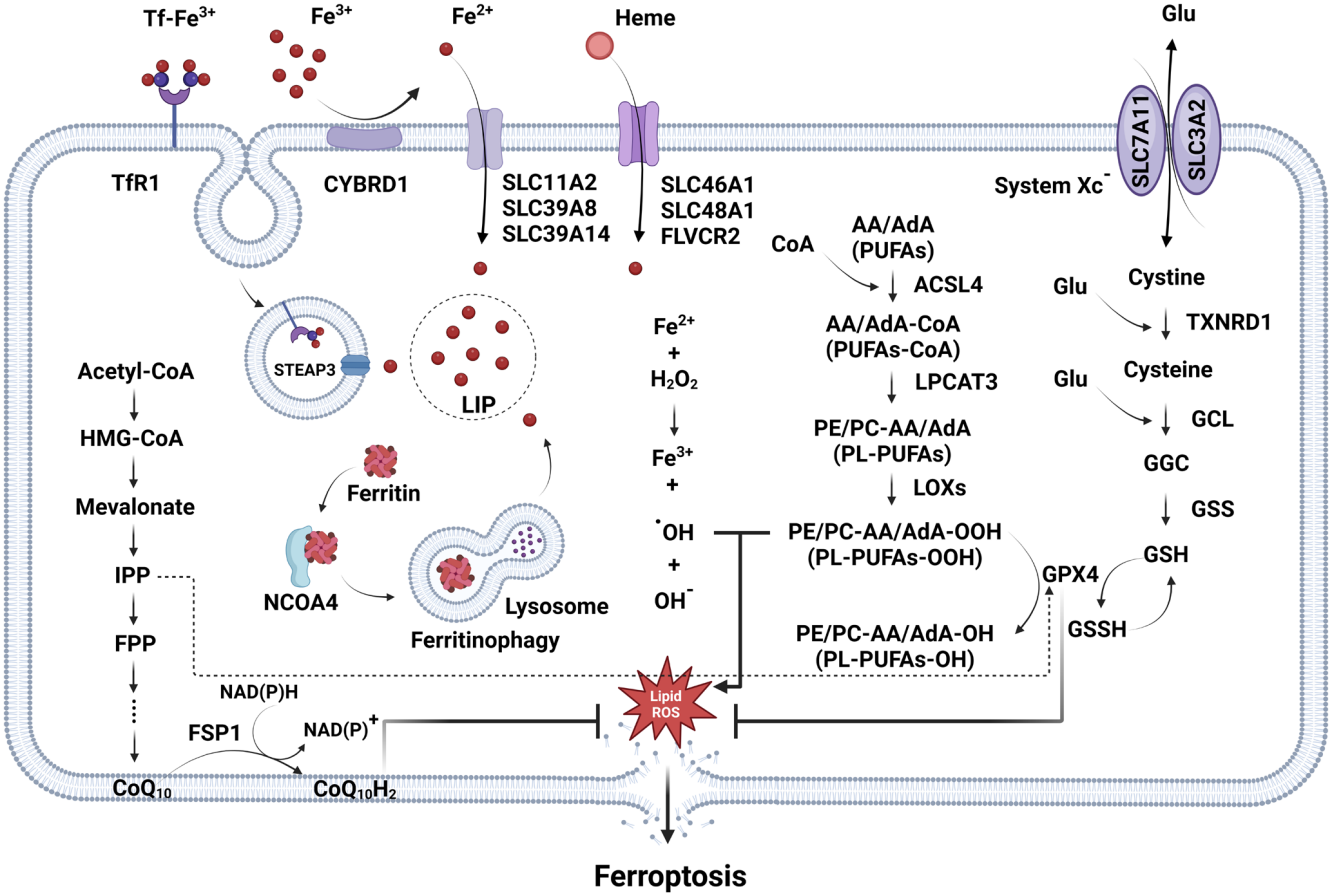
The ferroptotic cascade is regulated by iron metabolism, lipid peroxidation and cellular antioxidant systems. The accumulation of free iron initiates ferroptosis, with iron uptake primarily occurring via endocytosis of the Tf‐Fe^3+^/TfR1 complex. Internalised Fe^3+^ is reduced to Fe^2+^ by STEAP3 and transported into the cytosol by DMT1, forming the LIP. The LIP is highly active in the cytoplasm and contributes to cellular and lipid ROS generation, triggering ferroptosis. Iron homeostasis is maintained through ferritin (FTH) storage, utilisation as an enzyme cofactor and export via ferroportin. The autophagic degradation of FTH, referred to as ferritinophagy, is mediated by NCOA4, a cargo receptor that binds to the heavy chain of FTH, enabling delivery to the early autophagosome and release of iron into the cytoplasm. Another way to increase LIP is to release iron from heme. Phospholipids AA or AdA are crucial components of the cell membrane and are subject to esterification by the enzymes ACSL4 and LPCAT, resulting in the formation of PE‐AA/AdA. Subsequently, these compounds are further oxidised by LOXs to generate phospholipid hydroperoxides PE/PC‐AA/AdA‐OOH. Additionally, lipid peroxidation is also accelerated by iron‐dependent Fenton reactions, generating toxic lipid radicals. The intracellular antioxidant system neutralises lipid peroxides and their degradation products through GSH‐based redox reactions. The Xc^−^ antiporter (composed of SLC7A11 and SLC3A2 subunits) imports cystine while exporting glutamate. Intracellularly, cystine is reduced to cysteine, which combines with glutamate and glycine via GCL and GSS to produce GSH. GPX4, a key redox enzyme utilising GSH, reduces reactive lipid peroxides to alcohols. Additionally, CoQ_10_, a mevalonate pathway product reduced by FSP1, functions as a complementary radical‐trapping antioxidant, further mitigating ferroptosis.

**FIGURE 3:**
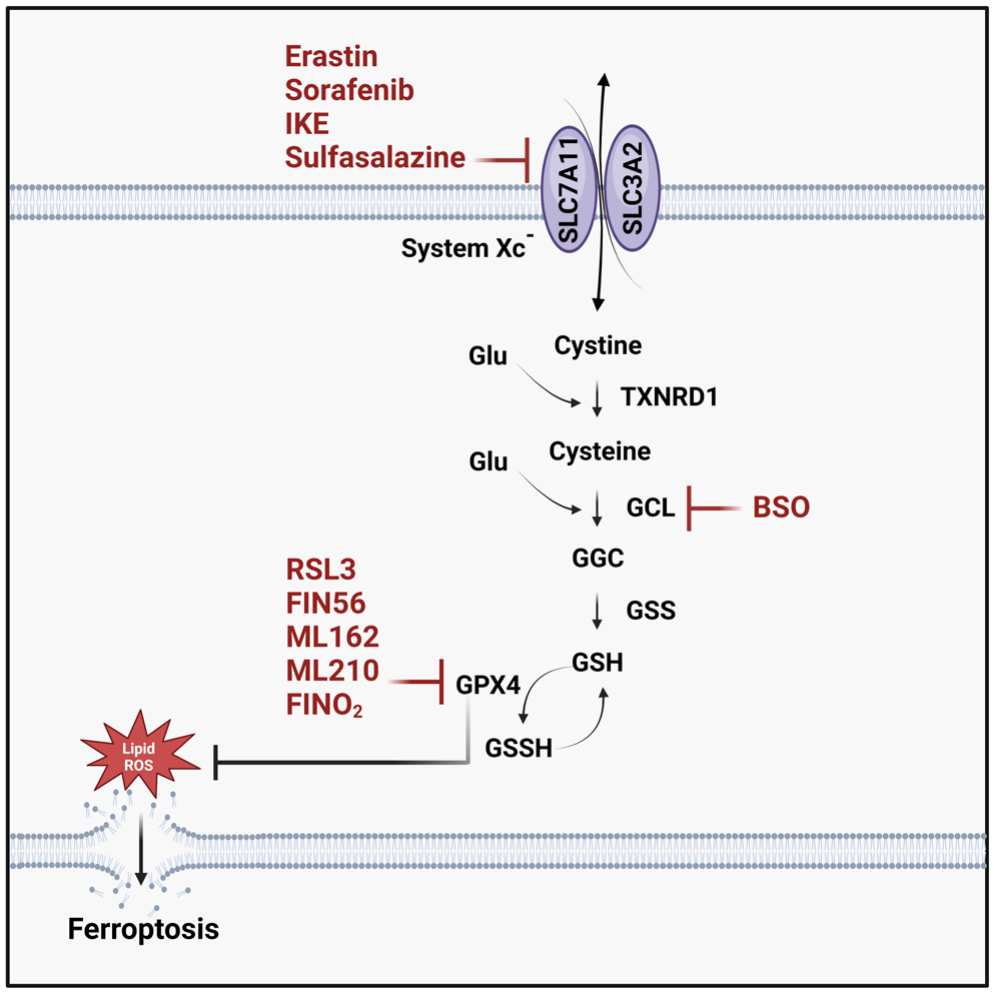
Ferroptosis inducers.

**FIGURE 4:**
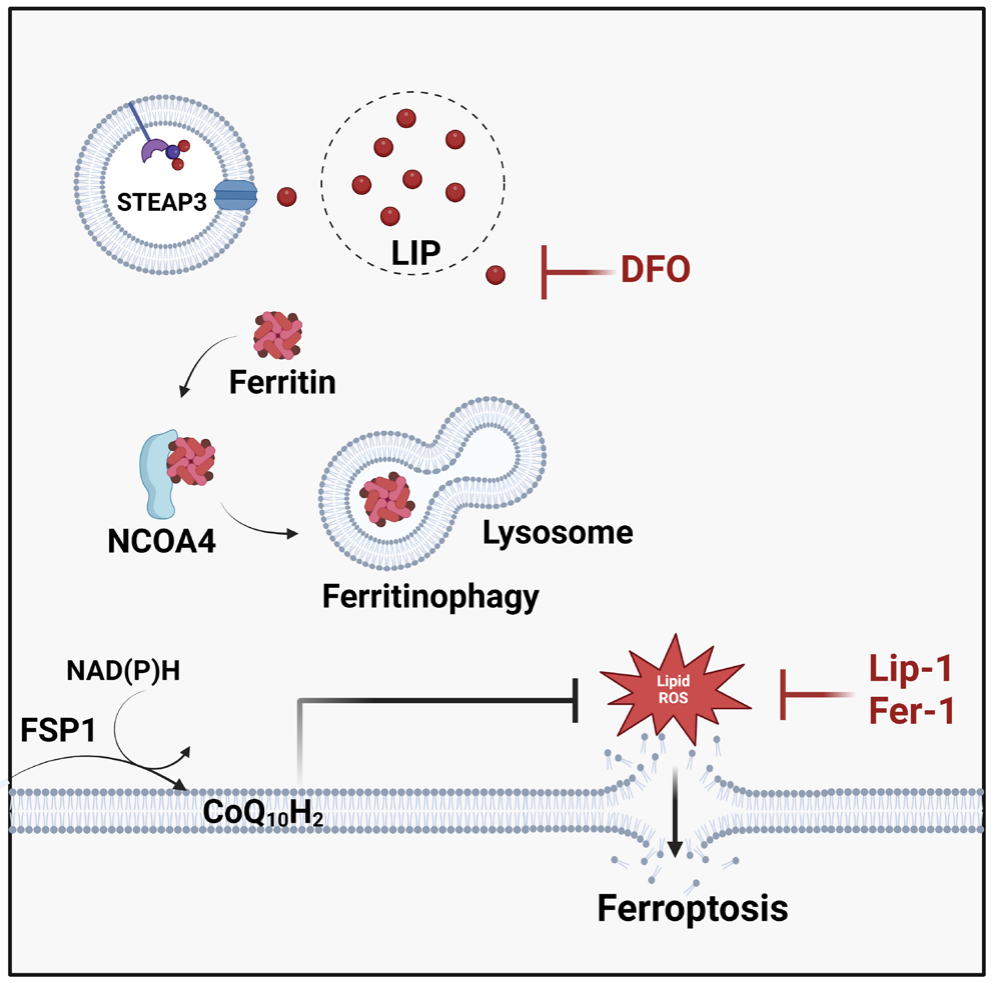
Ferroptosis inhibitors.

Cells rely on multiple antioxidant defence systems to counteract iron‐driven cytotoxicity. Disruption of the system Xc^**−**^‐Glutathione (GSH)‐GPX4 axis, which is involved in cellular antioxidant mechanisms, is one potential pathway that FINs may influence [32]. The system Xc^**−**^ consists of two subunits, SLC7A11 and SLC3A2, which facilitate the exchange of cystine and glutamate [33]. The SLC7A11 subunit performs the catalytic tasks, whereas the SLC3A2 subunit regulates the process [32]. The rapid reduction of cystine is initiated by thioredoxin reductase 1 (TXNRD1), which produces cysteine as a substrate for GSH biosynthesis [34, 35]. The GPX4 enzyme, along with its essential cofactor GSH, converts lipid hydroperoxides (L‐OOH) into alcohol form (L‐OH), thereby shielding the cell from ferroptotic damage [36]. Certain compounds known as FINs can trigger cell death through various mechanisms, such as indirectly inhibiting GPX4 by depleting GSH or directly inhibiting GPX4 [9]. Alternatively, ferroptosis suppressor protein 1 (FSP1) inhibits lipid peroxidation independently of GPX4 by facilitating the conversion of ubiquinone or Coenzyme Q (CoQ_10_) to ubiquinol (CoQ_10_H_2_) [37]. (Figures 1, 2, 3, 4).

## 3 Research Methodologies in Ferroptosis Research

Ferroptotic cell death is studied using methodological approaches that integrate pharmacological, genetic and analytical techniques. The most widely used strategy combines biochemical detection techniques, including C11‐BODIPY probes for lipid ROS measurement, FerroOrange for LIP assessment and GSH quantification to assess antioxidant status, alongside ferroptosis inducers and/or specific inhibitors such as Ferrostatin‐1 and Liproxstatin‐1 to confirm cell death [38]. (Figures 3 and 4) Genetic tools, including siRNA‐mediated knockdown and CRISPR‐based gene editing, are employed to disrupt key regulators of ferroptosis. In addition, quantitative real‐time PCR (qRT‐PCR) and sequencing are used to detect transcriptional changes. Mass spectrometry‐based proteomics is used to identify protein changes during ferroptosis. Immunoblotting tracks specific markers, while proximity labeling techniques map protein–protein interactions within ferroptosis regulatory networks [39, 40]. Morphological features of ferroptosis are assessed through live‐cell imaging with ferroptosis‐specific probes for real‐time monitoring and electron microscopy to reveal mitochondrial damage [8, 41]. Furthermore, in vivo studies using animal models verify ferroptosis mechanisms in real biological systems [42].

## 4 A Deep Insight Into Ferroptosis Susceptibility in AML

AML arises from genetic abnormalities in haematopoietic stem cells and excessive proliferation of immature cells [43]. It is most prevalent among individuals around 70 years of age and represents one of the most common forms of leukaemia, accounting for approximately 80% of adult leukaemia cases and about 3% of leukaemia cases in children under 15 years old [44, 45]. Ferroptosis, a type of iron‐dependent cell death, has emerged as a promising alternative therapeutic strategy for eradicating cancer cells, particularly those resistant to conventional treatments [46]. The following section focuses on the effects of various FINs on AML cells, categorised into four main groups: small‐molecule compounds, natural derivatives, synthetic agents and nanoparticles.

### 4.1 Small Molecules

Over the past decade, several small‐molecule compounds have been investigated for their potential to induce ferroptosis. Erastin, a classic ferroptosis inducer, exerts cytotoxicity by selectively inhibiting system Xc^**−**^ and depleting cysteine [47]. Research has provided evidence that erastin triggers cell death in HL‐60 NRAS‐Q61L cells, regardless of their RAS mutation status. Ferroptosis, apoptosis, autophagy and necroptosis are involved in the death process. It effectively induces ferroptosis, especially when combined with chemotherapeutics like cytarabine (Ara‐C) or doxorubicin (DOX), via activation of the c‐JUN N‐terminal kinase (JNK)/p38 pathway [48]. Interestingly, erastin treatment leads to the release and translocation of the high mobility group box 1 (HMGB1) transcription factor from the nucleus to the cytoplasm of HL‐60 NRAS‐Q61L cells. HMGB1 modulates erastin‐induced ferroptosis and necroptosis and regulates TfR1 expression through the RAS‐JNK‐p38 signalling axis. HMGB1 knockdown mitigates the cytotoxic effects of erastin and Ara‐C, reducing ROS production via the lysosomal iron‐dependent pathway [42]. Furthermore, erastin decreases the expression of ferritin heavy chain 1 (FTH1) and GPX4, effects that are counteracted by liproxstatin‐1 (Lip1) [49]. The serine protease inhibitor Kazal type 2 (SPINK2) knockdown heightens erastin sensitivity through p53‐mediated downregulation of SLC7A11 and iron metabolism [50].

Another small molecule, APR‐246, induces ferroptosis independently of TP53 mutational status by reducing GSH levels and increasing lipid ROS levels in AML cell lines. Tumour cells counteract APR‐246 exposure by increasing cystine uptake and upregulating SLC7A11 expression. Combining APR‐246 with erastin or GPX4 inhibitors (RSL3 and FINO2) has demonstrated synergistic effects on cancer cell death, enhancing the efficacy of ferroptosis induction [51].

RSL3, a direct GPX4 inhibitor, that exhibits potential antitumor properties through its ability to increase the content of intracellular iron and the lipid peroxidation product malondialdehyde (MDA). The function of RSL3 in iron‐catalysed lipid ROS production is enhanced by brain and muscle ARNT‐Like protein‐1 (Bmal1) gene silencing, which can be attributed to the suppression of early B‐cell factor 3 (EBF3) promoter methylation and the subsequent increase in arachidonate 15 LOX (ALOX15) expression via the Bmal1‐EZH2‐EBF3‐ALOX15 signalling axis [52]. The epigenetic regulator protein arginine methyltransferase 1 (PRMT1) knockdown enhances RSL3‐induced ferroptosis through ACSL1 upregulation, although in vivo responses remain inconsistent [38]. Nuclear factor erythroid 2 (Nrf2) inhibition via ML385 potentiates RSL3/FIN56‐induced effects through downregulation of antioxidant defences [53]. Also, Aldh3a2, an aldehyde‐detoxifying enzyme, contributes to ferroptosis resistance. Its inhibition in combination with RSL3 or chemotherapy drugs such as Ara‐C and DOX, results in significant synergistic effects by accumulating fatty aldehydes generated from lipid peroxidation [54].

The FSP1‐CoQ10‐NAD(P)H antioxidant system also suppresses ferroptosis independently of the GSH‐GPX4 axis [55], where ubiquinone prevents ferroptosis triggered by GPX4 inhibition in AML cells [56]. Disruption of this system via caseinolytic peptidase P proteolytic (ClpP) hyperactivation agents (ONC201 and ONC212) with ML210, a direct GPX4 inhibitor, exhibits potent inhibition of leukaemia cells. The ClpP complex functions within the mitochondrial matrix, and either inhibition or hyperactivation of ClpP decreases respiratory chain activity, leading to cancer cell death [57].

Furthermore, targeting glutamine metabolism via glutamate dehydrogenase 1 (GDH1) inhibition reduces glutamine uptake and Xc^−^ antiporter activity by downregulating SLC1A5 and SLC3A2. It is noteworthy that the addition of R162 and Ara‐C to GDH1 inhibition enhances cytotoxicity, emphasising glutaminolysis as a metabolic vulnerability [58].

### 4.2 Natural Compounds

Natural bioactive products and herbal medicines, recognised for their lower toxicity, have also shown promise in inducing ferroptosis in AML cells [59]. Dihydroartemisinin (DHA), derived from the Chinese plant Artemisia, induces apoptosis, autophagy and ferroptosis through activation of the AMP‐activated protein kinase (AMPK)‐mammalian target of rapamycin (mTOR)‐p70S6k pathway, reducing FTH1 expression and increasing iron levels, leading to autophagy‐dependent ferroptosis [60]. Moreover, DHA displays an anti‐ferroptotic effect on AML cells by upregulating metallothioneins (MTs), which are involved in zinc metabolism and GSH regeneration. Inhibition of MTs enhances DHA‐induced ferroptosis, particularly in combination with ferric ammonium citrate (FAC) [61].

Similarly, Typhaneoside (TYP), a flavonoid from *Pollen Typhae*, activates apoptosis signalling, reduces anti‐apoptotic proteins and induces autophagy‐mediated ferroptosis via the AMPK pathway triggered by ROS generation [62]. Sulforaphane (SFN), an isothiocyanate, is classified as a ferroptosis inducer class I (FIN I) and class II (FIN II) due to its direct inhibition of system Xc^−^ and its impact on GPX4 expression. AML cell lines with WT‐FLT3 (wild‐type‐FLT3) and FLT3‐ITD (FLT3‐internal tandem duplication) mutation exhibit apoptosis at lower SFN concentrations, while higher concentrations induce ferroptosis [63]. Honokiol, derived from the magnolia tree and known for its antioxidant and anticancer properties, demonstrates a statistically significant reduction of AML cell clone numbers and survival rates without harming normal blood cells. It increases lipid peroxidation in AML cells by upregulating the expression of heme oxygenase‐1 (HMOX1). However, the mechanisms behind the increase in SLC7A11 expression and the inability to decrease GPX4 expression after honokiol treatment warrant further investigation [64]. Perillaldehyde, a chemical from plants in the Sahara region, has been tested on AML and ALL cells and results show that it induces ferroptosis‐mediated cell death more effectively in AML cells. Perillaldehyde influences oxidative conditions by increasing lipid ROS levels while decreasing GPX4 and GSH expression. The mechanism of GPX4 inhibition by perillaldehyde requires further exploration, though it may exhibit partial selectivity for tumour cells [65]. Triptolide, derived from a Chinese tree, reduces Nrf2 expression in DOX‐resistant leukaemic cells with elevated Nrf2 levels. Decreased Nrf2 leads to reduced GPX4 expression, elevated ROS levels and heightened lipid peroxidation, ultimately causing ferroptosis. Combined treatment with triptolide and DOX shows synergistic anti‐leukaemic effects, particularly in drug‐resistant leukaemic cells [66].

### 4.3 Synthetic Compounds

Various synthetic chemicals have been found to induce ferroptosis by targeting redox balance, lipid metabolism or iron homeostasis. The all‐trans retinoic acid (ATRA)‐derived active compound, 4‐Amino‐2‐Trifluoromethyl‐Phenyl Retinate (ATPR), triggers autophagy‐mediated ferroptosis via downregulation of NCOA4 and FTH1, with suppression of antioxidant defences through Nrf2 inhibition [67]. Moreover, a synthetic iron(III) complex, chlorido [N,N′‐disalicylidene‐1,2‐phenylenediamine], along with a modified 4‐F component complex, promotes ferroptosis and necroptosis in leukaemic cells [68].

The combination of granulocyte colony‐stimulating factor (G‐CSF) and purified recombinant human thrombopoietin (rhTPO) in a study involving AML cell lines and mice results in more pronounced cell death compared to either agent alone. G‐CSF induces pyroptosis through neutrophil elastase, activating gasdermin D (GSDMD), while rhTPO induces ferroptosis by suppressing E1A binding protein P300 (EP300) binding to the GPX4 gene promoter and increasing MDA levels [69].

Neratinib, a tyrosine kinase inhibitor (TKIs), induces dose‐dependent ferroptosis marked by downregulation of GPX4 and FTH1. Autophagy inhibition diminishes ferroptosis, while subsequent erastin administration heightens the pro‐apoptotic effects of the cells [70]. Furthermore, resveratrol, a multiple kinases inhibitor, could intensify ferroptosis by downregulating the NFS1 and GPX4 expression levels through the hsa‐miR‐335‐5p/NFS1/GPX4. Also, the importance of NFS1 with cysteine desulfurase activity in regulating ferroptosis has been demonstrated; knockout of NFS1 is associated with an increase in iron uptake facilitated by TfR, an elevation in LIP and the production of ROS involved in ferroptosis [71]. Similarly, imetelstat, a telomerase inhibitor, heightens lipid peroxidation in AML cells by increasing polyunsaturated lipid content via ACSL4 and fatty acid desaturase 2 (FADS2), and its efficacy is improved following anthracycline/Ara‐C pretreatment [72].

Sulfasalazine (SSZ) inhibits the Xc^−^ antiporter and depletes GSH pools in AML cell lines dependent on cysteine metabolic pathways, resulting in significant survival reduction without apoptosis or autophagy. The nucleophosmin gene (NPM1)‐mutated AML cells with inherently higher cysteine‐related gene expression are more sensitive to cystine uptake inhibition. SSZ combined with the anthracycline daunorubicin (DNR) exhibits a significant synergistic effect in vitro, and it also enhances DNR/Ara‐C anti‐leukaemic activity in NPM1‐mutated AML primary samples and a patient‐derived xenograft (PDX) model [73]. Primary samples and different myeloid cell lines rely on cysteine, which cannot be synthesised from methionine, but L‐homocysteine (HCY) almost completely restores cystine depletion. Combining SSZ and buthionine sulfoximine (BSO) drives ROS production, hence limiting cell survival [74].

### 4.4 Nanoparticle‐Integrated FINs in AML

Nanoparticles coated with agents to target the molecular features of cancer cells have therapeutic potential by improving drug delivery [75]. The Fe_3_O_4_ nanoparticles (FeNPs) coated with 2,3‐dimercaptosuccinic acid (DMSA) exhibit the ability to diminish the viability of HL‐60 cells with lower CD^34+^ expression by enhancing iron levels and ROS generation through the Fenton reaction. Conversely, another iron‐based nanoparticle, Prussian blue nanoparticles (PBNPs), despite elevating iron content, fails to significantly reduce the survival of AML cells due to their ROS‐scavenging properties [76]. In a different approach, synthesised gold nanorods (GNRa)‐CSP12 are enhanced with chitosan and 12‐mer peptides to improve biodistribution and target leukaemic cells via TfR binding, induce ferroptosis and decrease N6‐methyladenosine (m^6^A) demethylase activity by disrupting the GSH‐GPX4 axis and altering the Fe^2+^/Fe^3+^ ratio in AML cells [77]. Likewise, GNPIPP12MA nanoparticles, designed to lower GSH levels and disrupt GPX4 function, effectively target m^6^A mRNA hypermethylation, decreasing mRNA transcript stability in AML cells [78].

Novel nanoplatforms known as GCMNPs, composed of a core of glycyrrhetinic acid and poly(lactic‐co‐glycolic acid) wrapped in a leukocyte membrane, effectively lower GPX4 expression in AML cells. These GCMNPs, when combined with ferumoxytol iron‐containing particles, accelerate ferroptosis via the Fenton reaction. Furthermore, a combination of GCMNPs, ferumoxytol and anti‐programmed death‐ligand 1 (anti‐PD‐L1) not only enhances the immune response but also hinders cysteine uptake by tumour cells, indirectly reducing GSH through T cells and thereby inducing ferroptosis [79].

Considering the dependence of AML cells on cholesterol uptake and their high expression of scavenger receptor B‐1, gold‐containing lipid nanoparticles (Au‐LNP) have been designed to target these receptors. This targeted approach reduces GPX4 expression, ultimately decreasing cell survival [80]. A GSH‐responsive cysteine polymer‐based ferroptosis‐inducing nanomedicine (GCFN) with a disulfide bond in the cystine structure makes GPX4 inactive by depleting reduced glutathione. Combining this FIN with the chemotherapeutic drug paclitaxel in GCFN (PTX@GCFN) effectively decreases the severity of leukaemic cells [81]. The zinc oxide nanoparticles (ZnO NPs) synthesised using hydrothermal processes increase the susceptibility of cells to ferroptosis by downregulating the expression of protective genes SLC7A11 and GPX4 while upregulating iron, ACSL4 and p53 [82] (Table 1).

**TABLE 1:** AML‐related FINs.

Type of FINs	FINs	Preclinical or clinical status	Synergism	Cell death	Marked effects	Crucial signalling pathway	Test model‐database	References
Small molecule	Erastin	Preclinical	Cytarabine Doxorubicin	Ferroptosis Necroptosis Autophagy Apoptosis	↑ p‐JNK, p‐p38 ↓ GPX4 ↓ Intracellular chelatable iron	JNK/p38	HL‐60 cell line	[48]
	Erastin		Cytarabine	Ferroptosis Necroptosis	↑ p‐JNK, p‐p38 ↑ Cytoplasmic HMGB1 ↓ GPX4 ↑ TfR1 ↑ PTGS2, MDA	RAS‐JNK/p38/TfR1	HL‐60 NRAS‐Q61L cell line NOD/SCID mice	[42]
	Erastin		SPINK2 inhibition	Ferroptosis	↓ SLC7A11, Cysteine ↑ Iron, STEAP3 ↑ ALCAM	NA	GDM‐1, KG‐1a, OCI‐AML3 and MOLM13 cell lines Primary samples Oncomine and NCBI GEO databases	[50]
	APR‐246	Phae Ib/II (NCT03931291, NCT03072043, NCT03588078)	Erastin RSL3/FINO2	Ferroptosis	↓ GSH ↑ SLC7A11	NA	MOLM‐14, OCI‐AML2, HL‐60, THP1 and SET2 cell lines Primary samples NSG mice	[51]
	RSL3	Preclinical	shBmal1	Ferroptosis	↓ Bmal1, EZH2 ↓ H3K27me3 ↑ EBF3 ↓ GSH, GPX4 ↑ Iron ↑ ALOX15, MDA	Bmal1/EZH2/EBF3/ALOX15	HL‐60 and NB4 cell lines Primary samples BALB/c nude mice	[52]

	RSL3 FIN56	Preclinical	PRMT1 inhibition	Ferroptosis	↓ H4R3me2a ↑ ACSL1 ↑ Iron	PRMT1/ACSL1	NB4, HEL and MOLM‐13 cell lines TCGA, GTEx and KEGG databases NSG mice	[38]
	RSL3 FIN56 Erastin	Preclinical	ML385	Ferroptosis	↓ Nrf2 (Following Nrf2 inhibition: SLC7A11, GCLC, GCLM, FTL, FTH1, HMOX1) ↓ GPX4	Nrf2/GPX4	MV4‐11, KG‐1a, KG‐1 and Kasumi‐1 cell lines Primary samples TCGA and KEGG database	[53]
	ML210	Preclinical	ClpP‐hyperactivation	Ferroptosis	↓ GPX4 ↑ mROS	NA	OCI‐AML3, OCI‐AML2, MOLM‐13 and HL‐60 RhoWT cell lines Primary samples TCGA dataset NSG mice	[57]
R162	Preclinical	Cytarabine	Ferroptosis	↑ GDH1 activity ↓ SLC1A5, SLC3A2 ↓ GSH, GPX activity, Cystine, Glutamine ↑ Glutamic acid	NA	MOLM‐13 and MV4‐11 cell lines Primary samples B‐NSG mice	[58]
Natural compound	DHA	Preclinical	Erastin	Ferroptosis Autophagy Apoptosis	↑ p‐AMPK ↓ p‐mTOR, p‐p70S6K, p‐S6 ↓ GSH, GPX4 ↓ FTH1, ISCU Ferritinophagy ↑ IRP2 ↓ MMP	AMPK/mTOR/p70S6k	HL60 and THP‐1 cell lines Primary samples BALB/c nude mice	[60]
	DHA		FAC MTs‐inhibitor	Ferroptosis	↑ MT isoforms ↑ SLC7A11 (Nrf2 activation) ↓ GSH ↑ LIP ↓ TfR1 ↑ FTH1 (Nrf2 activation), FTL, Tf, HMOX1	Zinc pathway	MOLM‐14 and OCI‐AML2 cell lines Primary samples KEGG database	[61]
					Co‐treatment with low doses of FAC: ↑ TfR1, LIP ↓ FTH1			
	Typhaneoside	Preclinical	NA	Ferroptosis Autophagy Apoptosis	↑ p‐AMPK ↓ p‐mTOR ↓ GSH, GPX4 Ferritinophagy ↓ FTH ↑ IRP2 ↓ MMP	AMPK/mTOR	Kasumi‐1, HL60 and NB4 cell lines Primary samples BALB/c nude mice	[62]
	Sulforaphane	Preclinical	NA	Ferroptosis Apoptosis	↓ GSH, GPX4 ↑ MDA	NA	U‐937 and MV4‐11 cell lines	[63]
	Honokiol	Preclinical	NA	Ferroptosis Apoptosis	↑ SLC7A11 ↑ HMOX1 ↑ SAT1	NA	THP‐1, U‐937 and SKM‐1 cell lines Primary samples NCBI GEO and KEGG database	[64]

	Perillaldehyde	Preclinical	NA	Ferroptosis	↓ GSH, GPX4 ↑ Extracellular ATP (ICD inducer)	PI3K‐ER‐Golgi	HL‐60 and Jurkat cell lines Primary samples	[65]
Triptolide	Preclinical	Doxorubicin	Ferroptosis	↓ GPX4, Nrf2	NA	HL‐60 and K562 cell lines Primary samples	[66]
Synthetised compound	ATPR	Preclinical	NA	Ferroptosis Autophagy	↓ GSH, GPX4 Ferritinophagy ↓ FTH1, NCOA4, Nrf2 ↑ MDA, COX‐2 ↓ MMP ↓ T‐SOD ↑ CD11b (Myeloid differentiation marker)	NA	HL60, U‐937 and NB4 cell lines NCG mice	[67]
	Chlorido iron (III) complex	Preclinical	NA	Ferroptosis Necroptosis	↑ mROS	NA	HL‐60 cell line	[68]
	rhTPO	Preclinical	G‐CSF	Ferroptosis Pyroptosis	↓ GPX4 ↑ Iron ↑ MDA	rhTPO/EP300	HL‐60 and KG‐1a cell lines Nude mice	[69]
	Neratinib	Phase I/II (NCT02932280)	Erastin	Ferroptosis Autophagy Apoptosis	↓ GPX4 ↑ Iron ↓ FTH1 ↑ ACSL4, MDA	NA	HL‐60 cell line	[70]
	Resveratrol	Preclinical	NA	Ferroptosis	↑ **Hsa‐miR‐335‐5p** ↓ NFS1 (Following NFS1 inhibition: ↓ GPX4, ↑ LIP, Tf) ↓ GSH	Hsa‐miR‐335‐5p/NFS1/GPX4	AML‐193 and OCI‐AML‐3 cell lines Primary samples TCGA, NCBI GEO and GTEx databases	[71]

	Imetelstat	Phase II (NCT05583552)	Doxorubicin Cytarabine	Ferroptosis Autophagy	↑ PUFA ↑ ACSL4, FADS2 ↑ Lipid droplet colocalisation, Vimentin (lipophagy)	NA	NB4, MV4‐11, KO52, HEL, MOLM13 and TF1 cell lines NCBI GEO database NSG, NSGS and NRGS mice (PDX model)	[72]
	Sulfasalazine	Phase I/II (NCT05580861)	Daunorubicin Cytarabine	Ferroptosis	↓ SLC7A11, GSH	BRD4/SLC7A11	IMS‐M2, OCI‐AML3 and MOLM‐14 cell lines Primary samples TCGA, NCBI GEO, GSEA, MSigDB and KEGG databases NSG‐SGM3 and hu NOG‐EXL mice (PDX model)	[73]
Sulfasalazine	BSO		Ferroptosis	↓ GSH, Extracellular‐Glutamate ↑ Extracellular Cystine, Glutamate	NA	MOLM13, MV4‐11, TF1, U937, KG1, OCI‐AML2, OCI‐AML3 and HL‐60 cell lines Primary samples	[74]
Nanoparticle	FeNPs	Preclinical	NA	Ferroptosis	↑ SLC7A11, GCLM, NQO1 ↑ Iron, FTL, DNM1 ↓ TfR ↓ GBA	HIF‐1 signalling way	HL‐60 and KG‐1a cell lines KEGG database	[76]
	GNRa‐CSP12	Preclinical	TKIs Anti‐PDL1	Ferroptosis	↓ m6A demethylase activity ↓ GSH, GPX4 ↓ Fe^2+^/Fe^3+^	GSH, Fe^2+^/FTO, ALKBH5/m^6^A/SLC2A3, CD276 and PKM (glycolysis, hypoxia and immune checkpoint pathways‐related genes)	NB4, MV4‐11 and Kasumi‐1 cell lines KEGG and GSEA databases Immunodeficient NOG mice	[77]

	GNPIPP12MA	Preclinical	Anti‐PDL1	Ferroptosis	↓ m6A demethylase activity ↓ GSH, GPX4 activity ↓ Fe^2+^/Fe^3+^ ↑ HMOX1 ↑ CD8^+^, CD4^+^, IFNγ	GSH, Fe^2+^/FTO, ALKBH5/m^6^A/MYC and CEBPA	Kasumi‐1 and MV4‐11 cell line KEGG and GSEA databases C57BL/6 mice	[78]
	GCMNPs	Preclinical	Ferumoxytol Anti‐PDL1	Ferroptosis	↓ GSH, GPX4 ↑ Iron ↑ HMOX1 ↑ CD8^+^, CD4^+^, IFNγ	NA	Kasumi‐1 and NB4 cell lines C57BL/6 J mice	[79]
	Au‐LNP	Preclinical	NA	Ferroptosis	↓ GPX4 ↑ CD11c, CD16, CD24 (Myeloid differentiation marker)	NA	MV4‐11, HEL and U937 cell lines Primary samples TCGA and GTEx databases	[80]
	GCFN	Preclinical	Paclitaxel	Ferroptosis	↓ GSH, GPX4 activity ↑ MDA	NA	MLL‐AF9 and THP‐1 cell lines C57BL/6J mice	[81]
ZnO NPs	Preclinical	NA	Ferroptosis Apoptosis	↑ p53 ↓ SLC7A11, GSH, GPX4, GPX activity ↑ Iron ↑ ACSL4, MDA	NA	NB4 and HL‐60 cell lines Primary samples	[82]

Abbreviations: ACSL1, acyl‐CoA synthetase long‐chain family member 1; ACSL4, acyl‐CoA synthetase long‐chain family member 4; ALCAM, activated leukocyte cell adhesion molecule; ALOX15, arachidonate 15‐lipoxygenase; AMPK, AMP‐activated protein kinase; ATPR, 4‐amino‐2‐trifluoromethyl‐phenyl retinate; Au‐LNP, gold‐containing lipid nanoparticles; Bmal1, brain and muscle ARNT‐like protein‐1; B‐NSG mice, NOD‐Prkdcscid II2rgtm1/Bcgenv mice; BRD4, Bromodomain‐containing protein 4; BSO, buthionine sulfoximine; ClpP, caseinolytic peptidase P proteolytic; DHA, dihydroartemisinin; DNM1, dynamin‐1; EBF3, early B‐cell factor 3; EP300, E1A binding protein P300; ER, endoplasmic reticulum; EZH2, enhancer of zeste homologue 2; FAC, ferric ammonium citrate; FADS2, fatty acid desaturase 2; FeNPs, Fe_3_O_4_ nanoparticles; FINs, ferroptosis inducers; FTH1, ferritin heavy chain 1; FTL, ferritin light chain; FTO, fat mass and obesity‐associated protein; GBA, glucosylceramidase beta/β‐glucocerebrosidase; GCFN, GSH‐responsive cysteine polymer‐based ferroptosis‐inducing nanomedicine; GCLC, glutamate‐cysteine ligase catalytic subunit; GCLM, glutamate‐cysteine ligase modifier subunit; GCMNPs, glycyrrhetinic acid‐based nanoplatforms; G‐CSF, granulocyte colony‐stimulating factor; GDH1, glutamate dehydrogenase 1; GEO, gene expression omnibus; GNRa‐CSP12, gold nanorods loaded with chitosan and a 12‐mer peptide; GPX4, glutathione peroxidase 4; GSEA, gene set enrichment analysis; GSH, glutathione; GTEx, genotype‐tissue expression; H3K27me3, histone H3 lysine 27 trimethylation; H4R3me2a, asymmetric dimethylation of histone H4 on arginine 3; HIF‐1, hypoxia‐inducible factor 1; HMGB1, high mobility group box 1; HMOX1, heme oxygenase‐1; hu NOG‐EXL, NOD.Cg‐Prkdcscid IL2rgtm1Sug Tg(SV40/HTLV‐IL3, CSF2) 10‐7Jic/Jic Tac (hu NOG‐EXL); ICD, immunogenic cell death; IFNγ, interferonγ; IRP2, iron regulatory proteins 2; ISCU, iron–sulfur cluster assembly enzyme; JNK, c‐JUN N‐terminal kinase; KEGG, Kyoto Encyclopedia of Genes and Genomes; LIP, Labile iron pool; m6A demethylase, N6‐methyladenosine demethylase; MDA, malondialdehyde; MMP, mitochondrial membrane potential; mROS, mitochondrial reactive oxygen species; MSigDB, molecular signatures databases; mTOR, mammalian target of rapamycin; MTs, metallothionein members; NA, not available; NCOA4, nuclear receptor coactivator 4; NFS1, NFS1 cysteine desulfurase; NQO1, NAD(P)H quinone oxidoreductase 1; Nrf2, nuclear factor erythroid 2; NRGS, NOD.Rag1−/−Il2Rg−/−/hIL3,CSF2,KITLG; NSG mice, NOD/SCID IL‐2 receptorᵞ‐chain‐null mice; NSGS, NOD/SCID/IL2gR−/−/hIL3,CSF2,KITLG; PDL1, programmed death‐ligand 1; PDX, patient‐derived xenograft; PI3K, phosphoinositide 3‐kinases; PRMT1, protein arginine methyltransferase 1; PTGS2, prostaglandin‐endoperoxide synthase 2; PUFA, polyunsaturated fatty acids; RAS, rat sarcoma; rhTPO, recombinant human thrombopoietin; SAT, spermidine/spermine N1‐acetyltransferase 1; SLC1A5, solute carrier family 1 member 5; SLC3A2, Solute carrier family 3 member 2; SLC7A11, solute carrier family 7 member 11; SPINK2, serine protease inhibitor Kazal type 2; STEAP3, six‐transmembrane epithelial antigen of the prostate 3; TCGA, The Cancer Genome Atlas; TF, transferrin; TfR1, transferrin receptor 1; TKIs, tyrosine kinase inhibitors; T‐SOD, total superoxide dismutase; ZnO NPs, zinc oxide nanoparticles.

## 5 The Crosstalk Between Specific ncRNAs and Ferroptosis in AML

The exceptional regulatory capacities of ncRNAs in cellular processes, including ferroptosis, have led to the development of ncRNA‐based therapeutic approaches [83]. The upregulation of non‐coding RNA LINC00618 post‐vincristine administration culminates in apoptosis and ferroptosis in leukaemic cells. This effect is attributed to the downregulation of SLC7A11 and the upregulation of BAX and activated caspase‐3 [84].

The introduction of the circRNA, CircKDM4C into myeloid cells, even when initially expressed at low levels, causes an increase in p53 levels by downregulating has‐let‐7b‐5p. This leads to elevated expression of ACSL4 and prostaglandin‐endoperoxide synthase 2 (PTGS2) and reduced expression of GPX4 and FTH1. This manipulation triggers the ferroptosis pathway and reduces the malignant proliferation and invasion of tumour cells. Notably, the combination of CircKDM4C overexpression and erastin treatment exhibits more potent growth inhibition compared to erastin treatment alone [85].

Another circRNA, has‐circ‐0015278, acts as a miRNA sponge to regulate FRG in FLT3‐ITD AML disease development [86]. High circZBTB46 expression is associated with disease aggravation in AML patients. This circular ZBTB46 enhances tumour cell proliferation by acting as a miRNA sponge and upregulating SCD, which protects cells from ferroptosis. CircZBTB46 knockdown increases AML cell sensitivity to RSL3, leading to intense ferroptosis, which is counteracted by ferrostatin‐1 (fer‐1) [87].

## 6 CML Cells' Susceptibility to Ferroptosis

CML is a specific type of myeloproliferative neoplasm characterised by the disruptive BCR‐ABL1 fusion gene [88]. Treatment with TKIs has shown efficacy, but relapses and drug resistance can occur, particularly in advanced stages or incomplete treatment [89]. Thus, novel treatment strategies are essential to address these issues.

Compounds like erastin, RSL3 and ML162 have demonstrated lethal effects on CML cells by inducing ferroptosis [90]. K562 cells expressing higher levels of the GATA‐1 s isoform exhibit greater susceptibility to RSL3, possibly due to elevated GPX4 expression, resulting in intensified lipid peroxidation and ferroptosis induction [91].

Imatinib‐resistant K562/G01 cells exhibit elevated ROS and MDA levels, reduced GSH levels and diminished GPX4 activity under cysteine‐deficient conditions. Interestingly, wild‐type K562 cells remain unaffected by GSH‐GPX4 inhibition through cysteine deprivation. However, the study revealed that cysteine depletion also decreases TXNRD1 activity in resistant cells, and knockdown of TXNRD1 restores ferroptotic sensitivity in K562 cells [92].

The utilisation of natural FINs offers promising prospects in several therapeutic applications. In the presence of DHA, K562 cells display reduced viability due to the regulation of the iron homeostasis–ROS–AKT–mTOR path in both sensitive and resistant K562 cells, particularly in multidrug‐resistant K562/adriamycin (ADM), thereby heightening their sensitivity to ADM [93]. Moreover, hydroalcoholic extract from an artemisia species called vulgaris (HEAV) activates lysosomal Ca^2+^ release into the cytoplasm, which is probably related to various cell deaths, including ferroptosis [94].

## 7 Boosting Ferroptosis Induction in MDS

Myelodysplastic syndrome (MDS) encompasses a diverse group of myeloid lineage disorders characterised by abnormal growth of haematopoietic stem cells, genetic anomalies, myelodysplasia and peripheral blood cytopenia. Unfortunately, MDS patients have a significant risk of progressing to AML [95]. The treatment of MDS poses challenges due to the array of chromosomal or molecular irregularities present and the limited selection of approved medications [96]. Therefore, exploring novel treatment strategies is crucial.

One functional mechanism underlying the cytotoxic effects of DEC in MDS involves the depletion of GSH and suppression of GPX4 activity, leading to enhanced ferroptotic sensitivity. The cytotoxic effects of DEC are augmented by combining it with erastin. This combination displays increased efficacy in bone marrow mononuclear cells (BMMNCs) specimens from high‐risk (HR) MDS patients. DEC induces cell death in both low‐risk (LR) and HR MDS patient‐derived cells. In LR cells, the main mechanisms are apoptosis and ferroptosis, while HR cells exhibit predominant mechanisms of ferroptosis and necroptosis [97].

## 8 The Molecular Interplay Between FINs and ALL

ALL is a haematological malignancy that originates from malignant lymphocytes in blood, BM and other sites and affects approximately 1.6 individuals per 100,000 in the United States [98]. Although current treatments demonstrate an 80%–90% response rate, 25%–35% of patients experience relapse [99]. To address this challenge, alternative forms of cell death, such as ferroptosis, are being explored. Exploring the mechanisms of cell death involves focusing on the interaction between small molecules and different signalling pathways.

### 8.1 Small Molecules

Ferroptosis can be pharmacologically induced in ALL cells using various small molecules that interact with redox‐sensitive and apoptosis‐regulating pathways. For example, the second mitochondrial activator of caspase (Smac) mimetics, such as BV6 and LCL‐161, which counteract inhibitors of apoptosis, trigger cell death influenced by molecular redox signalling [100]. Specifically, the utilisation of BV6 in combination with RSL3 induces ferroptosis, while the erastin/BV6 combination leads to oxidative cell death unrelated to iron [101]. Similarly, lowering antioxidant defences using erastin or BSO, a GSH synthesis inhibitor, combined with LCL‐161 enhances tumour cell killing [102].

Notably, B‐ and T‐ALL cell lines show greater sensitivity to RSL3 and BSO due to their reliance on the GSH‐GPX4 axis and low expression of FSP1. This low FSP1 expression results from DNA hypermethylation, which can be reversed by decitabine (DEC), a DNA methyltransferase inhibitor, and tert‐butylhydroquinone (tBHQ), enhancing Nrf2‐FSP1 interaction and reducing leukaemia cell susceptibility to RSL3 [103]. Furthermore, Nrf2 inhibition and upregulation of progestin and adipoQ receptor 3 (PAQR3) intensify RSL3‐ and erastin‐induced ferroptosis in ALL cells [104]. RSL3 also induces ferroptosis in apoptosis‐resistant FAS‐associated death domain (FADD)‐deficient ALL cells, suggesting that ferroptosis could bypass traditional apoptosis resistance [105].

Erastin treatment in T‐ALL cells generates ROS and activates the mitogen‐activated protein kinase (MAPK) pathway via inhibition of GPX4 and SLC7A11. Inhibiting p38 MAPK reduces, while inhibiting extracellular signal–regulated kinase (ERK1/2) enhances, erastin‐induced ferroptosis [106]. Additionally, combining an autophagy activator with erastin further amplifies ferroptosis, leading to increased cell death. Autophagy regulates erastin‐induced ferroptosis by preventing the ubiquitination of the voltage‐dependent anion channel 3 (VDAC3) following the downregulation of F‐box and WD repeat domain‐containing 7 (FBXW7), an E3 ligase targeting VDAC3 [107].

Beyond erastin and RSL3, ML162 has been shown to suppress the proliferation of Jurkat cells, with the lipophilic antioxidant ferrostatin‐1 (Fer‐1) counteracting the cytotoxicity of RSL3 and ML162, confirming ferroptosis involvement. Additionally, inhibition of tetrahydrobiopterin (BH4), an antioxidant maintained by dihydrofolate reductase (DHFR), alongside GPX4 inhibition in the presence of methotrexate, synergistically increases lipid hydroperoxide levels [108].

### 8.2 Natural Compounds

The naturally occurring saponin ardisiacrispin B, extracted from the fruit of Ardisia kivuensis Taton (Myrsinaceae), shows significant cytotoxicity in drug‐sensitive (CCRF‐CEM) and even drug‐resistant (CEM/ADR5000) leukaemia cells. Ardisiacrispin B and/or DOX cause iron‐dependent mortality and apoptosis in CCRF‐CEM cells, countered by ferroptosis inhibitors [109]. Epunctanone is also identified as a FIN in these cell lines [110]. One more natural compound of interest, hydnocarpin D, a bioactive flavonolignan compound, suppresses T‐ALL cell proliferation by inducing cell cycle arrest, apoptosis and autophagy‐mediated ferroptosis. Ferroptotic cells exhibit increased lipid ROS and reduced GSH and GPX4 levels, which are prevented by autophagy inhibition [111]. Poricoic acid A (PAA), a compound from Poria cocos mushroom, induces ROS‐dependent autophagy via AMPK‐mTOR signalling pathway activation and apoptosis in T‐ALL cells. PAA exposure increases intracellular iron and decreases GSH levels, as well as GPX4 expression, making T‐ALL cells susceptible to ferroptosis [112]. The realgar mineral, primarily composed of tetraarsenic tetrasulfide (As_4_S_4_), facilitates ROS production by disrupting mitochondrial function and glycolysis, resulting in p53 activation and subsequent apoptosis and ferroptosis [113] (Table 2).

**TABLE 2:** ALL‐related FINs.

Type of FINs	FINs	Preclinical or clinical status	Synergism	Type of cell death	Marked effects	Crucial signalling pathway	Test model	References
Small molecule	RSL3/BV6	Preclinical	NA	Ferroptosis	NA	NA	Jurkat and Moult‐4 cell lines	[101]
	RSL3	Preclinical	NA	Ferroptosis Necroptosis	↓ GPX4	NA	CTV‐1, Jurkat, MOULT‐16, MOULT‐13, REH cell lines CAM TARGET and TCGA databases	[103]
	BSO	Preclinical	NA	Ferroptosis Apoptosis	↓ GSH	NA	CTV‐1, Jurkat, MOULT‐16, MOULT‐13, REH cell lines TARGET and TCGA databases	[103]
	Erastin RSL3	Preclinical	PAQR3 overexpression	Ferroptosis Apoptosis	↓ Nrf2 ↓ GCLC, NQO1 ↑ Iron ↓ FTH1, HMOX1 ↑ MDA	PAQR3/Nrf2	CEM‐C1 and Jurkat cell lines Primary samples	[104]
	RSL3	Preclinical	NA	Ferroptosis	NA	NA	Jurkat, Jurkat FADD def and Moult‐4 cell lines	[105]
	Erastin	Preclinical	NA	Ferroptosis	↑ p38 MAPK, ERK, JNK ↓ SLC7A11, GSH, GPX4 ↑ MDA	p38 MAPK	Moult‐4 cell line	[106]
	Erastin	Preclinical	Autophagy activator	Ferroptosis Autophagy	↓ FBXW7 ↑ VDAC3	FBXW7/VDAC3	Jurkat, CCRF‐CEM, Reh, Nalm6 and Sup‐B15 cell lines UbiBrowser database NOD/SCID mice	[107]

RSL3	Preclinical	DHFR inhibition	Ferroptosis Necroptosis	↓ BH4 ↓ PUFA	α‐TOH/BH2/DHFR	Jurkat cell line REACTOME database	[108]
Natural compound	Ardisiacrispin B Doxorubicin	Preclinical	NA	Ferroptosis Apoptosis	↓ MMP	NA	CCRF‐CEM and CEM/ADR5000 cell lines	[109]
	Epunctanone Doxorubicin	Preclinical	NA	Ferroptosis Apoptosis	↓ MMP	NA	CCRF‐CEM and CEM/ADR5000 cell lines	[110]
	Hydnocarpin D	Preclinical	NA	Ferroptosis Autophagy Apoptosis	↓ GSH, GPX4 ↓ FTH1 ↑ ACSL4, CDO1	NA	Jurkat and Moult‐4 cell lines	[111]
	Poricoic acid A	Preclinical	NA	Ferroptosis Autophagy Apoptosis	↑ p‐AMPK ↓ p‐mTOR, p‐S6 ↓ GSH, GPX4 ↑ mROS ↑ IRP2 ↓ FTH ↑ MDA ↓ MMP	NA	Jurkat and ALL‐SIL cell lines Primary samples BALB/c Nude and NOD/SCID mice	[112]
	As_4_S_4_	Phase IV (NCT05682131)	NA	Ferroptosis Apoptosis	↑ p53 ↓ SLC7A11, GPX4 ↑ GSH ↑ Iron ↓ MMP	HK2/ROS/P53	Nalm‐6 and RS4;11 cell lines NOD/SCID mice KEGG database	[113]

Abbreviations: ACSL4, acyl‐CoA synthetase long‐chain family member 4; As_4_S_4_, tetraarsenic tetrasulfide; BH2, dihydrobiopterin; BH4, tetrahydrobiopterin; BSO, buthionine sulfoximine; CDO1, cysteine dioxygenase 1; DHFR, dihydrofolate reductase; ERK, extracellular signal–regulated kinase; FADD def, FAS‐associated death domain deficient; FBXW7, F‐box and WD repeat domain containing 7; FINs, ferroptosis inducers; FTH1, ferritin heavy chain 1; GCLC, glutamate‐cysteine ligase catalytic subunit; GPX4, glutathione peroxidase 4; GSH, glutathione; HK2, hexokinases 2; HMOX1, heme oxygenase‐1; IRP2, iron regulatory proteins 2; JNK, c‐JUN N‐terminal kinase; MAPK, mitogen‐activated protein kinase; MDA, malondialdehyde; MMP, mitochondrial membrane potential; mROS, mitochondrial reactive oxygen species; NA, not available; NQO1, NAD(P)H quinone oxidoreductase 1; Nrf2, nuclear factor erythroid 2; p‐AMPK, phosphorylated AMP‐activated protein kinase; PAQR3, progestin and adipoQ receptor 3; p‐mTOR, phosphorylated mammalian target of rapamycin; PUFA, polyunsaturated fatty acids; SLC7A11, solute carrier family 7 member 11; TARGET, therapeutically applicable research to generate effective treatments; VDAC3, voltage‐dependent anion channel; α‐TOH, α‐tocopherol.

## 9 The Identified Roles of ncRNAs in Regulating Ferroptosis in ALL

Investigations into ncRNAs in ALL have uncovered significant findings. For instance, circ‐0000745 is upregulated in ALL patients and cell lines. Knockdown of circ‐0000745 upregulates miR‐494‐3p, which enhances erastin‐induced ferroptosis. This complex interplay between circ‐0000745, miR‐494‐3p and neuroepithelial cell transforming 1 (NET1) significantly influences apoptosis, ferroptosis, glycolysis and cell cycle progression in malignant ALL cells [114]. These findings shed light on the potential role of ncRNAs in the pathogenesis of leukaemia and provide helpful insights for further research and potential therapeutic approaches.

## 10 Iron‐Related Cell Death in CLL

CLL is most commonly diagnosed in individuals around the age of 70, with an annual occurrence rate of 4.9 cases per 100,000 people [115]. Enhancing the response rates and treatment outcomes of CLL through ferroptosis induction in conjunction with current treatment methods holds promise.

Stromal cells supply CLL cells' cysteine requirements for GSH synthesis, which improves their survival, reduces cell death and contributes to drug resistance. Combining GSH‐depleting β‐phenylethyl isothiocyanate (PEITC) with an oxaliplatin chemotherapeutic drug shows synergistic effects in inhibiting CLL cells supported by stromal cells [116]. Additionally, malignant B lymphocytes display elevated levels of ROS, rendering them more vulnerable to ferric ammonium citrate (FeAC). The combination of FeAC with either ibrutinib or venetoclax demonstrates greater efficacy in killing CLL cells compared to individual treatments [117].

## 11 Ferroptotic Cell Death–Related Alterations in Lymphoma

Lymphoma encompasses a broad range of disorders originating from lymphocytes, with more than 90 subtypes. It is primarily classified into two main categories: Hodgkin lymphoma (HL) and non‐Hodgkin lymphoma (NHL), with the latter being more prevalent [118]. In the United States, its incidence rate is around 22 per 100,000 people [119]. The relapse rates for NHL differ depending on the specific subtype, with the most frequent subtype, diffuse large B‐cell lymphoma (DLBCL), which exhibits a 40% lifetime relapse rate [118]. Hence, there is a pressing requirement to create new medications with enhanced anticancer properties.

### 11.1 Small Molecules

Research on small‐molecule compounds reveals that erastin and RSL3 suppress DLBCL cell line proliferation by producing lipid peroxidation and activating ferroptotic cell death. DLBCL cells exhibit greater sensitivity than other haematological malignancies, such as AML and MM cells [9]. Enhanced GPX4 protein stability bound to the fifth member of heat shock protein family A (HSPA5) in DLBCL cells treated with an EZH2 (a subunit of histone methylation transferase) inhibitor leads to escape from possible ferroptosis induction in response to increased TfR1 levels. The combined therapeutic approach utilising erastin and EZH2i demonstrates a synergistic effect in promoting ferroptosis [120].

Additionally, imidazole ketone erastin (IKE), a potent analog of erastin, induces ferroptosis in DLBCL cells and in tumour‐bearing mice by inhibiting the Xc^−^ system. Long‐term exposure to IKE results in increased expression of SLC7A11, likely due to cysteine deficiency, but GPX4 expression remains unchanged. To enhance IKE's efficiency, polyethylene glycol‐poly (lactic‐co‐glycolic acid) (PEG‐PLGA) nanoparticles were utilised. These IKE‐loaded nanoparticles (IKE PEG‐PLGA NPs) exhibit improved quality and reduced off‐target toxicity and maintain similar antitumor activity [121].

One more agent, APR‐246, converted into methylene quinuclidinone (MQ), is capable of reactivating mutant TP53 by targeting specific cysteines in the core domain and refolding the protein. The DNA binding ability of the mutant TP53 is improved as well [122]. DLBCL cells carrying one missense mutation in TP53 and those that still have functional TP53 exhibit greater sensitivity than DLBCL cells with other TP53 mutations such as splicing, frameshift, nonsense or null TP53, demonstrating that TP53 is involved in APR‐246‐induced ferroptosis. Cells with a TP53 missense mutation located on exon 7 undergo a process called ferritinophagy‐mediated ferroptosis when exposed to APR‐246 in a TP53‐dependent manner. However, cells that have either the WT‐TP53 or other types of TP53 mutations experience ferroptosis [123]. APR‐246 appears to limit cell growth even when apoptosis and necroptosis are blocked in the absence of their mediators [124].

### 11.2 Synthetic Compounds

Studies on synthetic FINs, such as sulfasalazine (SSZ), have shown promise in limiting the growth of lymphoma cell lines by targeting the Xc^**−**^ transporter and cystine uptake. Although SSZ shows limited inhibitory effects in vitro on lymphoma cell lines lacking the Xc^**−**^ system, the inhibition of Xc^**−**^ in macrophage cells that supply cystine to lymphocyte cells significantly reduces tumour growth in vivo [125]. Moreover, SSZ at specific concentrations completely inhibits the growth of Xc^−^ transporter‐deficient lymphoma cells co‐cultured with cystine‐supplying fibroblasts [126].

Other substances act as follows: Altretamine, an FDA‐approved medication, directly inhibits GPX4 and enhances lipid‐OOH in DLBCL cells, providing an anticancer effect [127]. Dimethyl fumarate (DMF) inhibits the proliferation of DLBCL cells through the reduction of GSH and triggering ferroptosis due to its electrophilic properties. Since the germinal center B‐cell‐like DLBCL cells contain low levels of GSH and GPX4 and have high ALOX5 expression, they are more susceptible to DMF than activated B‐cell‐like DLBCL [128]. Furthermore, nanomolar concentrations of salinomycin‐derived ironomycin trigger the ferroptosis pathway by relocalising iron in lysosomes and activating the Fenton reaction, resulting in the overproduction of ROS. On the other hand, DLBCL cells treated with ironomycin display a significant decrease in GSH/GSSG ratios, while GSH levels remain approximately unchanged [129, 130].

Combination therapies also show promise. Co‐administration of a BCL‐2 inhibitor and a Bruton's tyrosine kinase (BTK) inhibitor downregulates BCL‐2 protein, upregulates cleaved caspase 3, depletes residual GSH levels and suppresses HMOX1, Nrf2 and GPX4 genes, contributing to apoptosis and ferroptosis in DLBCL cells, particularly in DHL cells [131]. Additionally, elevated glutamine levels and reduced alpha‐ketoglutarate (α‐KG) are associated with cell progression, with α‐KG likely being consumed in glutamine synthesis. The accumulation of α‐KG and its conversion to 2‐hydroxyglutarate (2‐HG) leads to ROS production and disrupts energy metabolism in dimethyl α‐KG‐treated DHL cells. Consequently, elevated ROS levels promote lipid peroxidation and TP53 activation, contributing to ferroptosis induction [132]. Combined treatment of [Au(d2pype)_2_]Cl, a thioredoxin inhibitor, which targets a critical component of the cellular antioxidant system, with ibrutinib, a BTK inhibitor, induces apoptosis in DLBCL cells. However, ferroptosis induction is dependent on the DLBCL subtype [133].

### 11.3 Natural Compounds

Several natural compounds have demonstrated potential in inducing ferroptosis across various lymphoma subtypes. One of the most well‐studied agents is artemisinin (ART), a FIN derived from a traditional Chinese medicinal plant [134]. ART activates the transcription factor 4 (ATF4)/CEBP‐homologous protein (CHOP)/cation transport regulator‐like protein 1 (CHAC1) pathway and increases CHAC1 gene expression with γ‐glutamyl cyclotransferase activity, leading to decreased GSH levels and reduced antioxidant capacity of Burkitt's lymphoma cells [135]. Another study has shown that ART inhibits signal transducer and activator of transcription 3 (STAT3) signalling in DLBCL cells, leading to cell cycle arrest and various subtypes of programmed cell death, including apoptosis, autophagy and ferroptosis. Additionally, data demonstrate that higher stimulation of ferroptosis and autophagy intensifies apoptosis [136]. As a combined treatment, ART and sorafenib have yielded encouraging outcomes in impeding the survival of NHL cells and promoting apoptosis and ferroptosis. These outcomes are accomplished through the downregulation of STAT3 and the reduction of GPX4 [137]. Also, HTLV‐1‐infected lymphocytes in adult T‐cell leukaemia/lymphoma (ATLL) cell lines undergo ferroptosis, necroptosis and apoptosis in response to ART [138].

Another natural compound, kayadiol, targets SLC7A11 and GPX4 expression through increasing p53 phosphorylation. The enhanced efficacy of kayadiol with cisplatin and/or asparaginase in reducing tumour cell viability is attributed to the activation of ferroptosis and overcoming chemotherapy resistance [139]. EBV‐infected NKTCL cells treated with DEC and gemcitabine undergo cell cycle arrest, apoptosis and ferroptosis. The expression levels of HMOX1 and SLC7A11 are increased and decreased, respectively, while GPX4 mRNA remains unchanged. However, it is worth noting that the GPX4 protein level is reduced [140]. Western honeybees process propolis, a substance that exhibits various biological activities such as antioxidant properties. It is able to alter protein expression, which illustrates that ferroptosis is the most prominent activated pathway in DLBCL cells [141].

### 11.4 Nanoparticle‐Integrated FINs

Currently, there is growing interest in the innovative application of nanoparticles in ferroptosis fields, including high‐density lipoprotein (HDL)‐like nanoparticles that obstruct cholesterol uptake by scavenger receptor type B1 (SCARB1) in B‐cell lymphoma cells. This leads to the activation of de novo cholesterol synthesis pathway genes and causes a considerable decrease in GPX4 expression [142]. According to the studies, iron oxide nanoparticles (IONs), which improve iron levels in patients with iron deficiency, have antitumour activities. In addition to elevated iron levels, they upregulate TfR expression and downregulate ferroportin protein, which leads to an increase in LIP. This process, combined with reduced GPX4 expression, makes DLBCL cells susceptible to ferroptosis and lipid peroxidation [143]. Furthermore, APP‐Fe NC nanocomplexes, which contain anti‐PD‐L1 peptides (APP), trigger Fenton's reaction and ultimately cause a disturbance in cellular redox balance in lymphoma cells. The combination of immunotherapy and ferroptosis induction achieved by APP‐Fe NCs effectively inhibits tumour growth in vivo and in vitro [144].

## 12 Triggering Ferroptosis in MM

MM is characterised by the accumulation of malignant plasma cells primarily within the bone marrow (BM), with potential progression and dissemination to other sites [145]. Despite significant advancements in MM treatment, many patients experience relapse, including those who initially respond well to therapy. Therefore, novel research is necessary to tackle recurrent and treatment‐resistant MM [146, 147].

Various FINs, such as erastin, RSL3 and ML162, have been shown to effectively target MM cells, regardless of glucocorticoid (GC) resistance. RSL3, in particular, induces changes in gene expression associated with ferroptosis and histone modifications in both GC‐resistant and GC‐sensitive cells [148]. Interestingly, the overexpression of ACSL4 in MM cells via the MAPK–ERK pathway is linked to increased cell proliferation. However, targeted knockdown of ACSL4 reduces cell proliferation and enhances susceptibility to ferroptosis, suggesting a potential therapeutic target [149].

To improve the therapeutic potential of current drugs, researchers are exploring novel approaches targeting the ferroptosis molecular pathways. The redox modulator BSO inhibits gamma‐glutamyl cysteine synthetase, leading to decreased GSH levels, which enhance the effects of bortezomib, a proteasome inhibitor, in MM therapy. The use of antioxidants offers protection in MM cell lines [150]. Another study has revealed that combining FeAC and bortezomib increases iron accumulation and MDA production in MM cell lines, showing a synergistic effect not seen with bortezomib alone [151]. Also, pretreatment of MM cells with omega‐3 PUFAs alters ferroptosis gene expression and GSH levels, enhancing susceptibility to subsequent bortezomib exposure [152].

Ultimately, natural compounds have been extensively investigated for their potential as FINs with therapeutic applications. Fingolimod (FTY720) induces ferroptosis, apoptosis and autophagy by targeting the protein phosphatase 2A (PP2A)‐AMPK‐eukaryotic elongation factor 2 (eEF2) pathway, leading to reduced SLC7A11 and GPX4 expression [153]. Chloroform from *Thymus vulgaris* and *Arctium lappa* plants [154], flavonoid apigenin [155] and *fumaria officinalis* extractions [156] stimulate cell death in MM cells, and the cytotoxic effects are mitigated by ferroptosis inhibitors, indicating ferroptosis involvement. Nitidine chloride, an extract from Chinese medicine, targets overexpressed ATP‐binding cassette transporter subfamily B6 (ABCB6) in MM cells, disrupting the PI3K‐AKT pathway and inducing ferroptosis [40]. The compound shikonin (SHK) triggers iron‐related death by downregulating SLC7A11 and GPX4 and inhibiting glutamic‐oxaloacetic transaminase 1 (GOT1), resulting in ferritinophagy [157]. Andrographolide regulates the p38–Nrf2–HMOX1 pathway, lowering Nrf2 and HMOX1 expression and elevating iron levels [158]. Furthermore, artesunate (ART) induces ferroptosis by promoting cytoplasmic localisation of sterol regulatory element binding protein 2 (SREBP2) and reprogramming MVA and GPX4 signalling. This is mediated by SREBP2–IPP–GPX4 [159].

## 13 Conclusions

Ferroptosis has emerged as a promising therapeutic strategy for treating haematological malignancies, particularly those that have developed resistance to standard treatments. This unique, iron‐dependent form of cell death takes advantage of cancer cells' increased sensitivity to oxidative stress and lipid peroxidation. In this review, we have explored how various FINs, ranging from small molecules and natural compounds to synthetic drugs and nanoparticle‐based agents, can reprogram metabolic pathways involved in iron handling, amino acid metabolism, ROS production and lipid regulation across diseases like leukaemia, lymphoma and multiple myeloma.

The increased vulnerability of these malignancies to ferroptosis appears to be linked to disruptions in FRGs and regulatory non‐coding RNAs, such as lncRNAs and circRNAs. Understanding these molecular circuits more deeply could help guide the development of targeted therapies, especially when used alongside conventional drugs to overcome treatment resistance. Notably, some FINs like APR‐246, Imetelstat, sulfasalazine and Neratinib are already being tested in clinical trials, and early results suggest they may work even better when combined with other ferroptosis‐inducing agents. That said, cancer cells are not defenceless. Many adapt by boosting cystine uptake and upregulating protective mechanisms like SLC7A11 or FSP1. These resistance pathways highlight the importance of finding reliable biomarkers and developing more personalised treatment strategies.

While the diseases covered in this review, AML, CML, CLL, lymphomas and others, are biologically diverse and treated in very different ways, they do share a common thread: they are all vulnerable, to varying degrees, to ferroptosis. For example, ferroptosis might not be relevant in early‐stage CML, which responds well to tyrosine kinase inhibitors, but could play a role in drug‐resistant or advanced cases. Similarly, CLL behaves more like an indolent lymphoma and may respond to ferroptosis under certain conditions.

In our view, the greatest promise lies in applying FINs to cancers that are particularly hard to treat, like those with TP53 mutations, resistance to apoptosis or high levels of oxidative stress. Advances in drug delivery technologies, such as nanoparticles or receptor‐specific carriers, could help target these agents more precisely and reduce potential side effects. Overall, ferroptosis represents a novel and exciting direction in cancer therapy. Going forward, it will be crucial to identify which patients are most likely to benefit, fine‐tune combination treatments and deepen our understanding of the molecular signatures that drive sensitivity or resistance to ferroptosis.

## Author Contributions

**Maryam Shayanmanesh:** investigation, writing – original draft. **Seyed Esmaeil Ahmadi:** investigation, writing – original draft, writing – review and editing. **Ali Amini:** conceptualization, investigation, project administration, supervision, visualization, writing – review and editing.

## Ethics Statement

This article does not contain any studies with human participants or animals performed by any of the authors.

## Consent

The authors have nothing to report.

## Conflicts of Interest

The authors declare no conflicts of interest.

## Data Availability

The authors have nothing to report.
